# Advanced MRI Assessment during Dendritic Cell Immunotherapy Added to Standard Treatment against Glioblastoma

**DOI:** 10.3390/jcm8112007

**Published:** 2019-11-17

**Authors:** Valeria Cuccarini, Domenico Aquino, Andrea Gioppo, Elena Anghileri, Serena Pellegatta, Carla Schettino, Federica Mazzi, Gaetano Finocchiaro, Maria Grazia Bruzzone, Marica Eoli

**Affiliations:** 1 Neuroradiology Unit, Fondazione IRCCS Istituto Neurologico Carlo Besta, 20133 Milan, Italy; valeria.cuccarini@istituto-besta.it (V.C.); domenico.aquino@istituto-besta.it (D.A.); andrea.gioppo@istituto-besta.it (A.G.); federica.mazzi@istituto-besta.it (F.M.); 2 Neuro-oncology Unit, Fondazione IRCCS Istituto Neurologico Carlo Besta, 20133 Milan, Italy; elena.anghileri@istituto-besta.it (E.A.); serena.pellegatta@istituto-besta.it (S.P.); carla.schettino@istituto-besta.it (C.S.); gaetano.finocchiaro@istituto-besta.it (G.F.); marica.eoli@istituto-besta.it (M.E.)

**Keywords:** Glioblastoma, Immunotherapy, Pseudoprogression, DSC-MRI, DWI-MRI

## Abstract

Evaluating changes induced by immunotherapies (IT) on conventional magnetic resonance imaging (MRI) is difficult because those treatments may produce inflammatory responses. To explore the potential contribution of advanced MRI to distinguish pseudoprogression (PsP) and true tumor progression (TTP), and to identify patients obtaining therapeutic benefit from IT, we examined aMRI findings in newly diagnosed glioblastoma treated with dendritic cell IT added to standard treatment. We analyzed longitudinal MRIs obtained in 22 patients enrolled in the EUDRACT N° 2008-005035-15 trial. According to RANO criteria, we observed 18 TTP and 8 PsP. Comparing MRI performed at the time of TTP/PsP with the previous exam performed two months before, a difference in cerebral blood volume ΔrCBV_max_ ≥ 0.47 distinguished TTP from PsP with a sensitivity of 67% and specificity of 75% (*p* = 0.004). A decrease in minimal apparent diffusion coefficient rADC_min_ (1.15 vs. 1.01, *p* = 0.003) was observed after four vaccinations only in patients with a persistent increase of natural killer cells (response effectors during IT) in peripheral blood. Basal rADC_min_ > 1 was independent predictor of longer progression free (16.1 vs. 9 months, *p* = 0.0001) and overall survival (32.8 vs. 17.5 months, *p* = 0.0005). In conclusion, rADC predicted response to immunotherapy and survival; Apparent Diffusion Coefficient (ADC) and Cerebral Blood Volume (CBV) modifications over time help differentiating PsP from TTP at onset.

## 1. Introduction

The development of immunotherapy against glioblastoma (GBM) has gained considerable interest over the last decade. Initial clinical trials showed increased overall survival in GBM patients treated by vaccines [1]; preclinical studies demonstrated activity of programmed cell death protein 1 inhibition in rodent glioma models [2], and checkpoint inhibitor drugs are now in clinical trials. Single-arm studies of Rindopepimut, a vaccine targeting Epidermal growth factor receptor variant III (EGFRvIII) in newly diagnosed EGFRvIII + GBM with minimal residual disease resulted in longer survival when compared to that of matched contemporary datasets, but the subsequent phase III study, ACTIV, failed to demonstrate a prolongation of survival by Rindopepimut when added to standard chemotherapy [3]. An unequivocal assessment of progression has become critical to evaluate the efficacy of immunotherapy in clinical trials. However, evaluating changes induced by immunotherapies on conventional brain magnetic resonance imaging (cMRI) can be difficult because such treatments may produce an inflammatory response that leads to increased contrast enhancement and vasogenic edema, similarly to GBM progression. Experience with Ipilimumab in non-central nervous system tumors demonstrated the occurrence of pseudoprogression (PsP): in some patients an early increase in enhancing tumor size or development of new enhancing lesions was followed by stabilization or later decrease of the enhancing tumor in the course of treatment [4]. In GBM treated with Nivolumab or Pembrolizumab with or without Ipilimumab, cMRI revealed an initial increase in enhancing volumes and edema within the first six months of immunotherapy [5]. To address this issue, new standardized criteria specific for immunotherapy were recently proposed (iRANO [6]). In particular, Immunotherapy Response Assessment in Neuro-Oncology (iRANO) proposed that patients with suspected PsP should be followed with serial MRI during the continuation of therapy for six months. Due to the short life expectancy of GBM patients, this approach remains problematic. Advanced MRI (aMRI) may allow for a deeper understanding of tumor structure and biology. Unlike contrast-enhancement, increased perfusion may be independent of disruption of the blood-brain barrier and defines microvascularity or neovascularity (angiogenesis) of the tumor. On diffusion-weighted imaging (DWI), the Apparent Diffusion Coefficient (ADC) inversely correlates to tumor cellularity and also yields information on potential white matter infiltration before these changes are visible on cMRI. 

In a previous paper, we reported clinical and immunological data of 24 patients affected by GBM and treated by Dendritic Cells (DC) immunotherapy, together with standard radio-chemotherapy, and we found that increased progression free (PFS) and overall survival (OS) were primarily associated with a significant, persistent activation of natural killer (NK) cells that was not detected in control patients from another parallel study conducted in our Institute that did not imply immunotherapy [7,8]. These data may be also sustained by other evidence supporting an important role of DC in NK priming based on IL-15 trans-presentation [9].

To evaluate the potential contribution of aMRI to distinguish the inflammatory response (i.e., PsP) from true tumor progression (TTP), and to identify patients who obtained therapeutic benefit from immunotherapy, we examined aMRI DWI and dynamic susceptibility-weighted imaging perfusion (DSC-MRI) findings of GBM at first diagnosis in patients treated with DC immunotherapy added to standard treatment (surgery, radiotherapy with concomitant chemotherapy with temozolomide (TMZ) followed by adjuvant TMZ) [10]. Nineteen patients were included in the previous study [8] and three were treated afterwards with the same protocol. Since GBMs are very heterogeneous tumors and given the presence of immune cells infiltrating the tumor during immunotherapy, the histogram analysis of ADC maps (which could allow a better representation of such heterogeneity [11]) was added to the identification of the Region of Interest (ROI). 

## 2. Experimental Section

### 2.1. Patients and Treatment Strategies

Patients who met criteria for participation in DENDR1 study (EUDRACT N° 2008-005035-15) were enrolled. Institutional Ethics Committee approved the study (protocol n. 419/2014), which conforms to the Declaration of Helsinki, and informed consent was obtained from all participants. 

All patients had histologically proven GBM, age ≥ 18 years and ≤ 70 years, neither multifocal nor sub-ependymal diffusion of the tumor, residual tumor volume after surgery < 10 cm^3^ confirmed by postoperative MRI assessment, Dexamethazone daily dose ≤ 4 mg during the 2 days prior to leukapheresis, Karnofsky performance score (KPS) ≥ 70. After surgery, patients underwent leukapheresis and radiochemotherapy, according to the Stupp protocol [10]. DC were loaded with whole tumor lysate and produced under Good Manufacturing Practices (GMP) conditions [12]. The first 4 vaccinations with tumor lysate loaded DC were performed every two weeks, from week 9 to 15. After the fourth vaccine, MRI was performed. Vaccinations 5 and 6 were spaced one month (week 19 and 23, respectively). The last vaccine dose (the 7th) was on week 31. The 1st, 5th, 6th and 7th vaccines contained 10 million DC; the 2nd, 3rd and 4th vaccines 5 million DC. Adjuvant TMZ started immediately after 3rd vaccination and continued for 6 cycles. 

**Figure 1:**
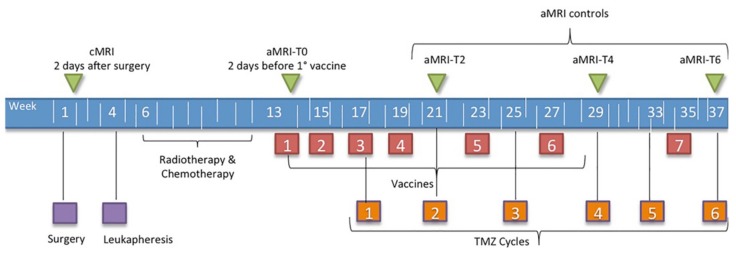
Schedule of treatment and follow-up time points. aMRI, Advanced magnetic resonance imaging; cMRI, conventional magnetic resonance imaging.

According to study protocol, patients underwent contrast enhanced cMRI within one week before surgery, within two days after surgery, and subsequently cMRI plus aMRI, including DWI and DSC-PWI within two days before the first vaccination, then every two months, or when clinical worsening occurred. Time points are displayed in Figure 1. At the same time, clinical monitoring was performed. Disease progression was defined according to RANO criteria [13], considering clinical performance, dose of steroids, and cMRI assessment as described in paragraph 2.4, using volume instead of diameters as suggested by Ellingson [14,15]. A patient showing signs of progression on imaging inside the radiation field during the first 12 weeks after completion of radiotherapy, was considered to have suspected tumor progression and maintained on the same treatment regimen and imaging follow-up, if clinically possible. PsP was defined as an increase of enhancing tumor volume ≥40% during the first six months of immunotherapy without significant clinical worsening and with stable or regressing lesions at the following MRI without changing therapy [6,15].

### 2.2. MRI Acquisition

MRI was performed using a Philips 3T scanner (Achieva TX; Philips Healthcare, Best, the Netherlands) with a 32-channel head-coil. The protocol included the following sequences: (i) 3D fluid attenuation inversion-recovery (FLAIR) (TR/TE = 4800 ms/333 ms, TI = 1650 ms, slice thickness = 1 mm, no gap, matrix = 240 × 240, Field Of View (FOV) = 240 × 240 mm); (ii) axial turbo spin-echo T2-weighted (TR/TE = 2313 ms/76.5 ms, Flip Angle (FA) = 90°, slice thickness = 3 mm, matrix = 1024 × 1024, FOV = 240 × 240 mm); (iii) single-shot echo-planar DWI (TR/TE = 2936 ms/62.5 ms, slice thickness = 4 mm, matrix = 288 × 288, FOV = 288 × 288 mm, 3 orthogonal directions, b = 0–1000 s/mm^2^, bicommissural acquisition) from which ADC maps were automatically reconstructed; (iv) DSC-PWI gradient-echo (GRE) (TR/TE = 1500 ms/40 ms, slice thickness = 5 mm, FA = 75°, matrix = 112 × 112, FOV = 224 × 224 mm, Gadovist®,0.1 cc/Kg, 5 mL/s and fixed 3 cc pre-bolus); (v) 3D-T1 fast-field-echo (FFE) (TR/TE = 9.93 ms/4.5 ms, FA = 8°, slice thickness = 1 mm, no gap, matrix = 240 × 240, FOV = 240 × 240 mm) before and after contrast-medium injection.

### 2.3. Post-Processing

#### 2.3.1. Volume Estimation

Tumor volumes (TV) were estimated on the 3D post gadolinium T1-weighted images by manually outlining the enhancing portion of the lesion using MRIcro ver. 1.4 (https://people.cas.sc.edu/rorden/mricro/mricro.html#Installation).

#### 2.3.2. Perfusion

DSC-MRI raw images were imported on a dedicated workstation where CBV maps were estimated. Maximum CBV (CBV_max_) was obtained by identifying regions of maximal perfusion from color maps. A senior neuroradiologist placed three ROI on the highest color areas of tumor. CBV values were normalized (rCBV) compared to an identical ROI positioned on the contralateral healthy white matter (CHWM). rCBV_max_ was obtained from the highest value within the ROI. 

#### 2.3.3. Diffusion

ADC maps were analyzed using two different approaches: (i) a classical ROI-based approach; (ii) a semi-automatic approach.

In the ROI-based approach, a senior neuroradiologist placed three identical circular ROIs in different areas of the lesion and one in the CHWM. The ROIs were placed on the basis of cMRI and ADC map appearance. The tumoral ADC minimum (ADC_min_) values were normalized (rADC) to those obtained from the CHWM.

Because of the heterogeneous cellularity of GBM, particularly during immunotherapy, mean modifications in ADC values from selected areas (ROIs) could not be as meaningful as ADC scale analysis of whole tumor volume; therefore, we decided to also analyze ADC distribution. In the semi-automatic approach, all the 3D-T1 scans and ADC maps were imported onto a dedicated workstation. The T1 volumes were then co-registered to the ADC maps using SPM12 (Statistical Parametric Mapping, https://www.fil.ion.ucl.ac.uk/spm/software/spm12/). After that, using a semi-automatic custom-made software, the contrast-enhancing volume was segmented on the registered T1 volumes. The software provides thresholds of the 3D-T1 images and requires the selection of one voxel of the tumoral volume to isolate it from the other regions. An expert neuroradiologist chose the threshold and seed voxel of each scan. The contrast enhancement volumes extracted from the co-registered T1 images were imported into Matlab (https://www.mathworks.com) and used as a binary mask on the ADC maps. The ADC histogram of each extracted enhancing volume was calculated, and from that the following statistic moments were estimated: (a) mean; (b) mode; (c) kurtosis; (d) skewness.

### 2.4. RANO Criteria 

RANO criteria including cMRI, corticosteroids use, and clinical status were published in 2010 to address the issues of pseudo-effects in radiochemotherapy or antiangiogenic therapy [13]. To overcome limitations of previous criteria, T2/FLAIR assessment of the lesions were included. Specifically: radiological partial response is defined as a decrease by ≥50% from baseline in the sum of products of the two major diameters of up to five target lesions (i.e., enhancing lesions with at least two perpendicular diameters of solid enhancing tissue both ≥1 cm); progressive disease as an increase by ≥25% from nadir (i.e., smallest seen) in the sum of product of diameters of target lesion or new lesions out of the radiation field or substantial worsening in T2/FLAIR (not due to edema, ischemia, demyelination, gliosis). Pseudoprogression after radiotherapy is considered if recurrence is present in the radiated field within 12 weeks after completion of radiotherapy and requires a repeated scan after 4 weeks to confirm or exclude progression. 

The iRANO committee redefined the response assessment criteria for patients with brain tumors undergoing immunotherapy [6]: in patients with early findings suggesting progression including new lesions within the first 6 months of immunotherapy regimen without substantial neurological decline, therapy should be continued and confirmation of radiographic progression by follow-up imaging should be sought 3 months after the initial radiographic evidence of progressive disease. 

Current RANO criteria are based on two-dimensional measurements on MRI. However, volumetric measurement would be more accurate and is encouraged. A diameters-volume conversion was suggested [15]: ≥25% increase in the sum of products of biperpendicular diameters of enhancing tissue means ≥40% increase of volume, while ≥50% decrease in the sum of products of diameters means means ≥65% volume decrease, respectively.

### 2.5. Immune Monitoring

Immune monitoring was performed on the whole blood of each patient before the treatment, after each vaccination, and every two months until tumor recurrence. 

T-cell subsets were monitored by flow cytometry using anti-CD3-VioBlue, anti-CD4-FITC and anti-CD8-APC and anti-CD56-PE monoclonal antibodies (Miltenyi Biotec). Briefly, 100 μL of whole blood was incubated with 10 μL of conjugated primary antibodies for 10 min at 4 °C. Acquisition analyses were performed using a MACSQuant analyzer and MACSQuantify Software ver. 2.13 (Miltenyi Biotec, https://www.miltenyibiotec.com/CA-en/products/macs-flow-cytometry/software/macsquantify/trial.html). An acquisition gate for lymphocytes was defined according to the side scatter vs. forward scatter parameters, and 5000 events.

The activation of immune cells (NK and CD8+ T cells) was evaluated as intracellular Interferon Gamma (IFN)-γ expression assessed by flow cytometry and as IFN-γ secretion detected by ELISA, in the PBLs of patients co-cultured with matched loaded-mature DCs. The strategy used to define the immune cell activation is described in detail in our previous paper [8], and representative dot plots of flow cytometry assessment of active NK cells and CD8+ T cells are displayed in the Supplementary Materials (Figure S1).

The ratio of the mean of vaccinations (2nd to 7th)/baseline values (V/B ratio) of absolute count and frequency of NK cells, CD8C and CD4C T cells for each patient was calculated, and the median of all of the observations was used as the cut off value to separate patients into the “low” or “high” groups. The threshold able to separate patients with “low” or “high” V/B ratio and having the best sensitivity and specificity, was defined using Receiver Operating Characteristic (ROC) curves.

### 2.6. Statistical Analysis

The following radiological parameters were collected for each patient at different time points until tumor progression: contrast-enhancing TV, rCBV_max_, rADC_min_ and semi-automatic ADC_mean_, ADC_mode_, ADC_skewness_.

Statistical comparison between radiological, immunological and clinical parameters was assessed using the Wilcoxon-Mann-Whitney tests. The Wilcoxon signed rank test was used to determine the significance of differences between radiological parameters at various time points. All *p* values were two-sided.

PFS was calculated from the first surgery until disease progression and death/last follow-up, if censored. OS was calculated from surgery to death due to any cause or last follow-up (censored). The Kaplan-Meier analysis was used to estimate PFS and OS. The log rank test assessed differences in progression or survival in patients with different radiological or clinical parameters. 

Multivariate analysis and Cox proportional hazard regression model analysis were performed on variables showing statistically significant differences at univariate analysis to investigate their independent prognostic role.

Receiver Operating Characteristic (ROC) curves were estimated to determine for TV, rCBV_max_, rADC_min_, ADC_mean_, ADC_mode_ and ADC_skewness_ the value of optimal sensitivity and specificity to differentiate patients in HighNK and LowNK (as defined in the Results paragraph), or to distinguish TTP from PsP.

All statistical analyses were performed using SPSS 22.0 for IBM (SPSS Inc., Chicago, IL, USA) software. 

## 3. Results

### 3.1. Clinical Data and Conventional MRI Assessment

Twenty-two patients in the DENDR1 study (EUDRACT N° 2008-005035-15) had analyzable data and were included in the imaging follow-up until tumor progression. Patients were divided into two groups based on their immune responses induced by DC vaccination. Thirteen patients with a significant, persistent activation of NK cells were defined HighNK patients, and nine patients without NK cell increase during immunotherapy were defined as LowNK. Patients with high NK cell count showed a significant and persistent activation of NK cell response and activation. The V/B ratio calculated as previously described in the text was correlated with PFS and OS, and the Kaplan Meier Curves (Figure S2) were used to display a significant correlation between high NK V/B ratio and better prognosis (prolonged survival): median PFS 17.2 vs. 9.3 months in HighNK vs. LowNK, *p* = 0.0003; median OS 32.8 vs. 12.5 months, respectively, *p* = 0.0001.

Time points of treatment and radiological follow-up are displayed in Figure 1.

Median age, gender, Karnofsky performance score (KPS), post-surgery TV did not significantly differ in the two subgroups, percentage of hypermethylation of the O(6)-methylguanine-DNA methyltransferase (MGMT) promoter in tumor was higher in HighNK patients (*p* = n.s.) (Table 1).

A significant difference in Median TV values was observed only at MRI obtained four months after immunotherapy start (i.e. after six vaccinations and three cycles of adjuvant TMZ, aMRI-T4): it was 3.3 cm^3^ (0–13.57) in HighNK patients and 7.5 cm^3^ (0.71–20.72) in LowNK (*p* = 0.04).

During the study, no partial responses were observed; 8 PsP and 18 TTP were noticed during the follow-up. Using ROC curves (Area Under the Curve (AUC) 0.70 *p* = 0.04) a threshold basal volume ≤ 5.63 cm^3^ was a significant predictor of longer PFS (15.4 vs. 9 months *p* = 0.028); the difference did not reach statistical significance for OS (29 vs. 17.5 months).

### 3.2. Advanced MRI Response Assessment and Stratification of Survival

#### 3.2.1. Response Assessment 

During the follow-up we observed 18 TTP (in 11 HighNK and 7 LowNK) according to RANO criteria (i.e., taking also into account clinical performance and steroid dosing). In 16 patients volumetric increase of contrast-enhancing lesion was observed, two of them had also leptomeningeal dissemination and two multifocal progression. Nine patients had a second surgery: in all, pathology revealed extensive areas with viable tumor cells.

**Figure 2:**
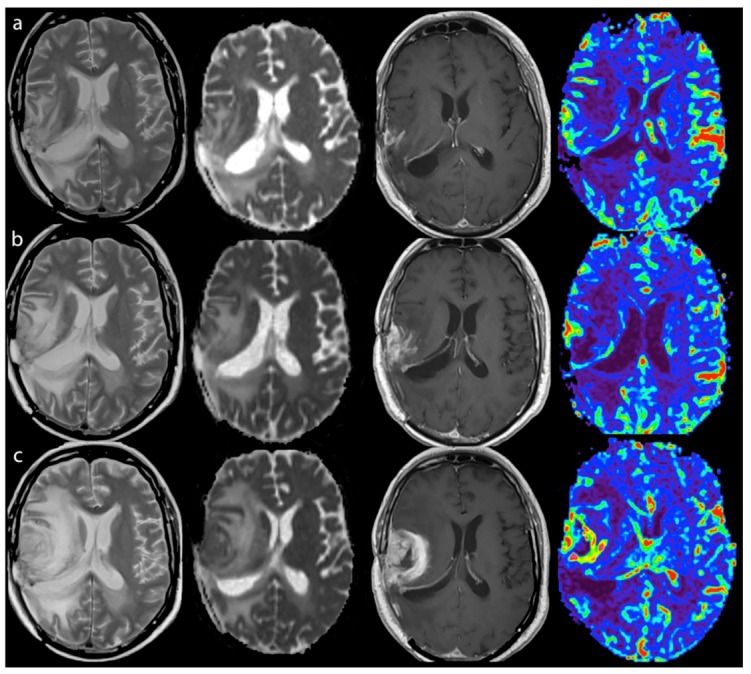
True tumor progression during immunotherapy of a LowNK and Unmethylated MGMT patient - Left to right: T2, ADC map, T1-enhanced and CBV map. (**a**) Oct 2013 after surgery and radio-chemotherapy and first four vaccinations, MRI-2 mo (Steroid dose 3 mg Dexamethazone, clinical condition stable): small GBM residual showing contrast enhancement (T1) and a spot of hyper-perfusion with high CBV (red to green), moderate edema and post-actinic alteration (T2 hyper-intensity) and slight ADC restriction; (**b**) Jan 2014, MRI-6 mo during immunotherapy (Steroid dose 3 mg Dexamethazone, clinical condition stable): enlargement of enhancing, of hyper-perfused and of ADC restricted volumes and of edema; (**c**) Mar 2014, MRI-8 mo after immunotherapy (Steroid dose 8 mg Dexamethazone, clinical condition worsening): further extension of enhancement with thick ring pattern due to central necrosis, more intense and wide hyper-perfusion and ADC restriction of the enhancing lesion, increased edema with mass effect. CBV, Cerebral Blood Volume; ADC, Apparent Diffusion Coefficient; MGMT, O (6)-methylguanine-DNA methyltransferase.

Comparing MRI performed at the time of TTP with the previous exam performed two months earlier, a significant increase of median rCBV_max_ (3.98 to 5.87, *p* = 0.03), and a significant decrease of rADC_min_ (1 to 0.93, *p* = 0.03) were observed (Figure 2). A trend to increased median ADC_skewness_ was also noted (Table 2).

**Figure 3:**
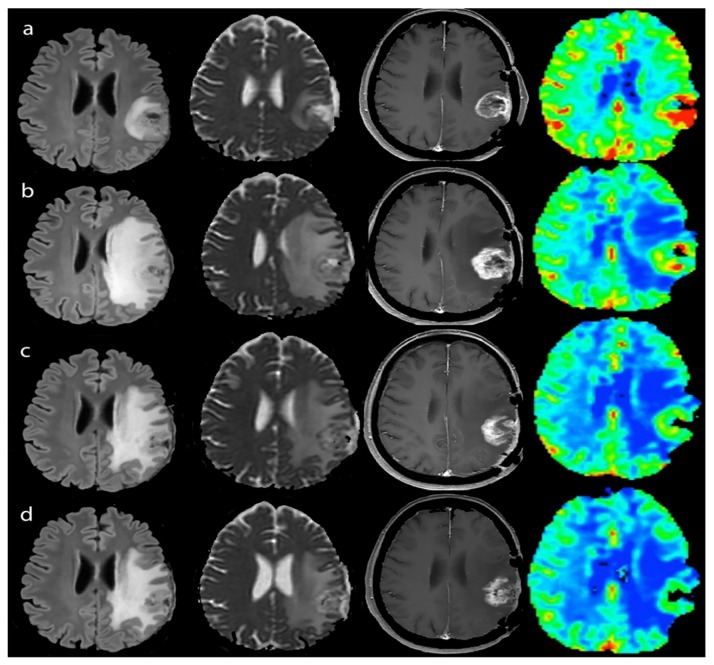
Pseudoprogression during immunotherapy of a LowNK and hypermethylated case - Left to right: FLAIR, ADC map, T1-enhanced and CBV map. (**a**) Jan 2015, MRI-pre, after surgery and radio-chemotherapy (steroid dose 2 mg Dexamethazone, clinical condition stable): GBM showing contrast enhancement (T1) and hyper-perfusion with high CBV (red-colored), slight edema (FLAIR) and non-homogeneous ADC restriction as in hypercellularity; (**b**) Mar 2015, MRI-2 mo during immunotherapy (no steroid therapy, clinical condition stable): enlargement of enhancing volume and of edema with mass effect and reduction of hyper-perfusional intensity (yellow to green CBV with small red spots) and persistent ADC un-homogeneity; (**c,d**) Jun and Aug 2015, MRI-4 and -6 mo after immunotherapy (no steroid therapy clinical condition stable): reduction of enhancing volume, edema and CBV intensity (green) and volume of hyper-perfusion with less restricted ADC.

During the study, 8 patients (4 HighNK and 4 LowNK, two with hypermethylated MGMT and six with unmethylated MGMT) experienced PsP. The events occurred two months after starting immunotherapy (aMRI-T2) in three patients, and after four months (aMRI-T4) in the other five cases. After initial tumor growth, patients diagnosed with PsP remained radiologically stable (Figure 3) according to RANO [6,14] without changes in therapy for an average 9.4 months (3.8–25.7), and they were able to taper steroids during the follow-up. 

A trend to reduction of median rCBV_max_ and ADC_skewness_ was noted comparing pre-PsP and PsP values. A significant decrease in median ADC_mode_ value (1.28 vs. 1.14, *p* = 0.012) was observed comparing values at PsP and at the following exam (Table 2), suggesting the presence of fluid accumulation (i.e., tumor edema) at the time of PsP.

ROC curves were estimated to determine the value of optimal sensitivity and specificity of all radiological parameters differentiating TTP and PsP. A rCBV_max_ > 3.91 was able to differentiate TTP from PsP with a sensitivity of 92.9% and specificity of 57.1% (AUC 0.73 *p* = 0.07); a rADC_min_ value ≤ 0.92 discriminated TTP and PsP with a sensitivity of 43.7% and specificity of 87.5% (AUC 0.6, *p* = 0.15). Statistically significant results were obtained when changes in rCBV were analyzed: an increase in rCBV_max_ ≥ 0.47 (i.e., MRI-TTP or PsP minus MRI-preTTP or PsP) discriminated TTP from PsP with a sensitivity of 67% and specificity of 75% (AUC 0.81, *p* = 0.007).

No difference in PFS and OS was observed between patients that experienced PsP and those who did not.

#### 3.2.2. Survival

After a median follow-up of 22.5 months, median PFS was 15.4 (95% C.I 9.5–21.3) in HighNK and 10.2 (95% C.I 7.6–12.8) months in LowNK (*p* = 0.006); median OS 32.8 (95% C.I 15.9–49.6) and 19.4 (95% C.I 14.1–24.6 *p* = 0.01) months. Clinical and immunological data of 19 of these patients were reported in a previous manuscript [8]. 

The analysis conducted via ROI did not show statistically significant differences at MRI performed soon before immunotherapy start (see Table 3). After the first four vaccinations rADC_min_ decreased in HighNK only (1.15 vs. 1.01 *p* = 0.003), while remained unchanged in LowNK; the decrease observed in HighNK persisted during the first four months of immunotherapy but not at aMRI-T6. ROC curves were estimated to determine the value with optimal sensitivity and specificity to differentiate the patients who developed a persistent increase of NK cells in peripheral blood during immunotherapy from those who did not: a basal rADC_min_ threshold >1 differentiated the two subgroups with a sensitivity of 100% and specificity of 75% (AUC 0.85 *p* = 0.002). rADC_min_ at aMRI-T2 also differentiated the two subgroups (Tables S1 and S2). A basal rADC_min_ > 1 was significant predictor of longer PFS (16.1 vs. 9 months, *p* = 0.0001) and OS (32.8 vs. 17.5 months, *p* = 0.0005).

The analysis with the semiautomatic method showed higher whole tumor ADC_mode_ values in High vs. LowNK patients (1.22 vs. 0.810^−3^ mm^2^/s, *p* = 0.03) at baseline MRI. In LowNK patients only ADC_mode_ and ADC_mean_ values increased during the first four months of therapy. No significant correlations between the other parameters, including perfusion metrics, and the outcome were observed.

**Figure 4:**
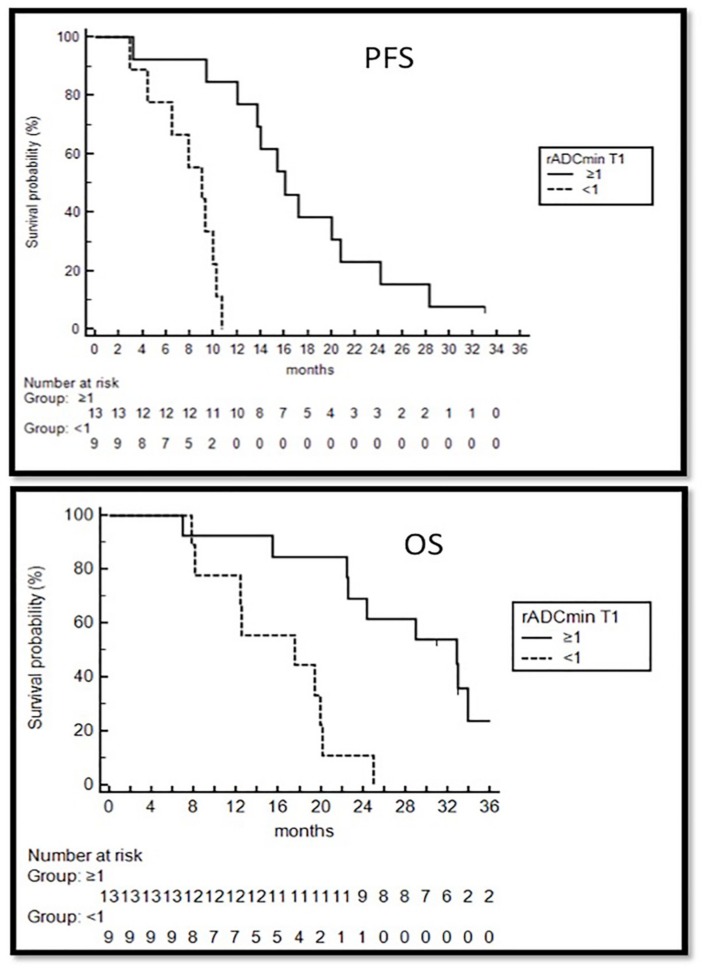
Survival analysis. Kaplan Meier curves showing progression free survival (PFS) and overall survival (OS) of patients with ADC ≥ 1 or ADC < 1 in the tumor at pre-vaccine MRI.

Multivariate analysis using parameters showing statistically significant differences in univariate analysis confirmed that basal rADC_min_ was the only independent variable affecting both PFS (Exp(b) 13.4 *p* = 0.001) and OS (Exp(b) 6.47, *p* = 0.002) (Figure 4).

## 4. Discussion

An accurate and timely identification of true tumor progression can be particularly difficult in GBM patients when immunotherapy is added to standard care. In immunotherapy, inflammatory reaction within and around the tumor is expected much more than with cytotoxic therapies. The enlargement of pre-existing enhancing lesions and the appearance of new enhancing lesions due to inflammation have been already described [16] and a clinical benefit in patients with an initial apparent progression has been reported [6,17].

GBMs are generally composed by different structural and functional regions, mixed scenarios with coexistence of glioma and treatment alterations are often the rule and multimodal treatments increase brain tissue heterogeneity. Thus, cut-offs in a single shot examination hardly distinguish between TTP and PsP and the evaluation of longitudinal modifications of parameters is recommended. Two are the main approaches to analyze MRI data: (a) the histogram approach in which the evolution of the whole lesion during therapy is quantitatively characterized estimating the statistical parameters of the distribution of the values inside the lesion; (b) the ROI-based approach, that aims to detect markers in specifically selected areas. The use of ROIs to evaluate the ratios with respect to the contralateral portion is a common clinical practice, being easy to perform and not requiring specific softwares to be analyzed. Our decision to use the classical ROI method to evaluate the role of CBV and ADC in the differentiation between PsP and TTP might be a limitation because of the gaps of the sample and the user-dependence of the delineation process. In our work, however, we reduced the effect of this potential bias by using multiple ROIs of identical size (in all MRIs of all subjects, within lesions and contralateral healthy brain); moreover, we specifically placed them in the same areas trying to choose the sites of both impaired perfusion and hypercellularity on CBV and ADC maps, albeit targeting different biologic processes. Furthermore, due to the particularly heterogeneous cellularity (tumoral and immune) during immunotherapy, mean modifications in ADC values from selected areas could not be as meaningful as ADC scale analysis of whole tumor volume; therefore, we decided to also analyze ADC distribution within a single whole tumoral ROI. 

Both PsP and TTP were observed in our study (PsP rate was 36.4%) within the first four months of immunotherapy, when according to iRANO criteria treatment should be continued besides radiological suspected progression in the absence of clinical worsening [6]. Subsequently TTP only was found. The two conditions are identical on cMRI and, in absence of clinical worsening, currently PsP or TTP are retrospectively dated depending on the clinical-radiological evolution. In our study, the comparison between MRI at TTP or PsP with the previous exam showed increase of rCBV_max_ and reduced rADC_min_ in TTP, while substantially stable rADC_min_ and a trend to rCBV_max_ and ADC_skewness_ reduction in PsP. Specifically, a difference (Δ) in cerebral blood volume ΔrCBV_max_ ≥ 0.47 was able to distinguish the two conditions with a sensitivity of 67% and specificity of 75% (*p* = 0.004).

Several studies have applied DSC-MRI to identify TTP. A wide spectrum of radiation-related modifications ranges from early subacute imaging changes to late radionecrosis [18]. 

CBV has been shown to differentiate with high accuracy tumor recurrence from late-delayed progressive enhancing following radiation therapy [19,20,21]; its potential for distinguishing PsP from TTP in the setting of early delayed progressive enhancement is more controversial [22,23].

In a recent meta-analysis including only patients who were treated by radiotherapy with concomitant and adjuvant TMZ developing a new lesion up to six months, Patel confirmed the usefulness of rCBVmax and rCBVmean to distinguish PsP from recurrent tumor reaching pooled sensitivity and specificity within the range of 80–90%, but the difficulty in defining a widely applicable threshold value was highlighted [24]. 

Longitudinal modifications in rCBV could be more useful than absolute rCBV in distinguishing PsP from TTP. Our results show that statistically significant results were obtained only when changes at a two-month interval in rCBVmax were analyzed. Boxerman reported similar results in patients with high grade gliomas treated with TMZ, paclitaxel poliglumex and concurrent radiation [25]. In tumors already treated with radiotherapy, viable tumor cells, inflammatory cells and necrosis may coexist with different vascular morphology and a potentially wide range of vascular volumes [26]. 

Previous studies have reported a difference in ADC values of PsP and TTP after radiochemotherapy but with different ADC thresholds [27,28,29]. In a pilot study of 8 recurrent GBMs treated with DC immunotherapy, highest rCBV and lowest rADCmin in the contrast–enhancing area were associated with tumor progression, but no data on longitudinal modification of those parameters and their relationship with treatment response were provided [30]. Quin, in a group of recurrent GBMs receiving immune checkpoint inhibitors described an initial increase in volume of tissue with intermediate ADC suggesting hypercellularity within the first six months of treatment and subsequent stabilization in patients who derived therapeutic benefit [5]. In our study, we observed in patients who had an immunological response with NK cells increase after immunotherapy a decrease in rADCmin and ADCskewness increase (describing greater frequency of low ADC levels) within the first four months of treatment. The result supports the hypothesis that hyper-cellularity subtended by lowering ADC may be explained by infiltrating immune cells rather than by tumor cells proliferation in progressive gliomas. Thus, ADC has to be concomitantly evaluated with CBV. The significant decrease in ADCmode observed at the MRI following PsP agrees with the hypothesis of fluid accumulation (i.e., tumor edema) at the time of apparent progression. 

In our patients, rADCmin and TV detected soon after radiochemotherapy were significant predictors of longer PFS; multivariate analysis showed that rADCmin was an independent prognostic factor with positive impact on the outcome. 

Ellingson has already proposed to use the first post-radiation MRI as baseline scan in clinical trials, instead of using the postoperative MRI [31].

The MGMT methylation status is one of the most relevant biomarkers predicting benefit from alkylating agent chemotherapy in GBMs. In one immunotherapy study it did not affect the outcome [8] and the differences in rADCmin and TV observed at first post-radiation MRI were not due to MGMT methylation status in our study (Table S3).

The ADC is inversely correlated with cell density, probably due to reduced water mobility from dense cellular packing [32]. In our study, basal rADCmin was significantly higher in HighNK than in LowNK and TV was lower. A mathematical model of malignant glioma treated by alloCTL (CD8+ cytotoxic T-cell) immunotherapy describing the quantitative interactions of six components tumor cells, CTLs IFNgamma and TGFbeta Major Histocompatibility Complex (MHC) class I receptor on a tumor cells and MHC class II receptor on antigen presenting cells has already been proposed [33,34]. As suggested by Agur the development of new methodologies for personal predictions of therapy outcomes, by the integration of patient data with dynamical mathematical models, is needed [35]. 

## 5. Conclusions

Pre-immunotherapy rADC resulted predictive of progression free and overall survival, highlighting the relevance of the ratio between cancer cell and immune cell number. Moreover, rADC_min_ reduction and ADC_skewness_ increase within the first months of therapy without significant CBV increase, indicated and predicted response to immunotherapy. The analysis of modifications over time in ADC and CBV can help differentiating PsP from TTP at onset during immunotherapy with dendritic cells.

## Figures and Tables

**Table 1:** Patients’ characteristics.

	HighNK	LowNK
N°	13	9
F/M	3/10	3/6
Age	54.7 (36–70)	54.7 (45–62)
KPS	90	90
MGMT Hypermethylation	6/13	1/9
Post-Surgery TV	0.77 (0–2.1)	1.74 (0.2–3.83)

KPS, Karnofsky performance score; MGMT, O(6)-methylguanine-DNA methyltransferase; TV, Tumor volumes; F/M, female/male.

**Table 2:** (**a**) median values with range observed two months before and during TTP. (**b**) median values with range observed two months before, during and two months after PsP.

**(a)**					
**TTP**					
	**MRI_preTTP_**		**MRI_TTP_**		**Δ (MRI_TTP_-MRI_preTTP_)**
Vol (cm^3^)	5.66 (0.24–25.83)		12.34 (1.26–38.31)		6.02 (0.5–29.5)
rCBV_max_	3.98 (0.87–9.28) ^a^		5.87 (2.81–15.18) ^b^		2.32 (−0.81–8.29)
rADC_min_	1 (0.81–1.59) ^c^		0.93 (0.67–1.32) ^d^		−0.14 (−0.62–0.26)
ADC_mean_ (10^−3^ mm^2^/s)	1.4 (1–1.7)		1.4 (0.8–1.7)		0.03 (−0.5–0.3)
ADC_mode_ (10^−3^ mm^2^/s)	1.2 (0.2–2)		1.1 (0.06–2.5)		−0.07 (−0.96–0.3)
ADC_skewness_	1.43 (0.93–3.77)		1.68 (1.05–10.9)		0.03 (−0.1–1.52)
**(b)**					
**PsP**					
	**MRI_prePsP_**	**MRI_PsP_**	**MRI_postPsP_**	**Δ (MRI_PsP_-MRI_prePsP_)**	**Δ (MRI_postPsP_-MRI_PsP_)**
Vol (cm^3^)	5.44 (0.45–9.41)	7.70 (0.60–20.72)	5.41 (0.4–13.7)	2.88 (0.25–14.15)	−0.89 (−6.96–0.72)
rCBV_max_	5.6 (1.85–14.14)	3.91 (0.75–10.04)	4.31 (2.97–7.63)	−2.64 (−5.35–3.68)	0.4 (−1.91–6.88)
rADC_min_	1.1 (0.81–1.43)	1.01 (0.82–1.34)	1.1 (0.88–1.01)	−0.09 (−0.27–0.2)	−0.01 (−0.22–1.18)
ADC_mean_ (10^−3^ mm^2^/s)	1.42 (1–1.7)	1.49 (0.9–1.66)	1.52 (1–1.6)	0.07 (−0.2–0.3)	−0.03 (−0.07–0.1)
ADC_mode_ (10^−3^ mm^2^/s)	1.22 (0.3–1.5)	1.28 (0.3–1.8) ^e^	1.14 (0.2–1.6) ^f^	0.6 (−0.3–0.5)	−0.2 (−0.5–−0.1)
ADC_skewness_	1.77 (1.15–2.75)	1.47 (1.18–2.09)	1.56 (1.1–3.3)	−0.33 (−1.5–0.15)	−0.09 (−0.36–2)

Note: a,b, *p* = 0.03; c,d, *p* = 0.03; e,f, *p* = 0.012.

**Table 3:** Median values with range observed before (MRI-T0) and during immunotherapy in High and Low NK patients.

	High/LowNK	aMRI-T0	aMRI-T2	aMRI-T4	aMRI-T12
Median TV T1 CE (cm^3^)	HighNK	3.2 (0–11.2)	2.71 (0–38.34)	3.3 (0–13.57) ^a^	0.94 (0–8.3)
	LowNK	5.7 (0.86–25.22)	6.6 (1.1–27)	7.5 (0.71–20.72) ^b^	-
rCBV_max_	HighNK	5.29 (0.56–10.55)	3.29 (0.92–14.14)	3.79 (0.76–10,04)	2.6 (0.71–6.89)
	LowNK	5.25 (2.27–9,69)	3.91 (1.81–7.52)	4.85 (1.08–6.82)	-
rADC_min_	HighNK	1.15 (0.82–1.47) ^c^	1.01 (0.76–1.51) ^d^	0.97 (0.67–1.34) ^e^	1.02 (0.85–1.32)
	LowNK	0.97 (0.69–1.61)	1 (0.82–1.47)	0.99 (0.7–1.11)	-
ADC_skewness_	HighNK	1.38 (0.99–3.15)	1.62 (0.84–2.75)	1.59 (0.71–2.1)	1.79 (1.07–4.47)
	LowNK	1.95 (0.85–2.44)	1.98 (1.4–2.3)	1.73 (1.4–2.35)	-
ADC_mode_ (10^−3^ mm^2^/s)	HighNK	1.22 (0.3–2) ^f^	1.24 (0.06–1.6)	0.8 (0.2–1.5)	1.1 (0.9–1.2)
	LowNK	0.8 (0.4–1.1) ^g^	1.24 (0.9–1.8) ^h^	1.3 (0.2–1)	-
ADC_mean_ (10^−3^ mm^2^/s)	HighNK	1.4 (1–1.7)	1.4 (0.8–1.7)	1.4 (0.9–1.8)	1.2 (0.9–1.9)
	LowNK	1.3 (1.1–1.6) ^i^	1.4 (1.3–1.6) ^l^	1.5 (1.2–1.7) ^m^	-

Note: a,b, *p* = 0.04; c,d, *p* = 0.003; c–e, *p* = 0.005; f,g, *p* = 0.03; g,h, *p* = 0.018; i–l, *p* = 0.018; i–m, *p* = 0.017.

## Supplementary Materials

The following are available online at https://www.mdpi.com/2077-0383/8/11/2007/s1, Figure S1: Flow cytometry assessment of active NK cells and CD8+ T cells; Representative dot plots show: (A) peripheral blood lymphocytes (PBLs) identified using Forward versus Side Scatter (FSC and SSC), based on size and granularity (complexity); (B) the gating strategy used to identify (1) CD45+ CD3- and (2) CD3+ CD45+ subset used to detect (C) CD56+ NK cells (Gate 1) and (D) CD8+ T cells (Gate 2) intracellularly stained with anti-IFN-g antibody; Figure S2: Meier curves display a significant correlation between high NK V/B ratio and prolonged survival; *** means *p* < 0.001; Table S1: Normalized CBV and ADC values before and during immunotherapy; Table S2: CBV and ADC values with range observed two months before, during and two months after PsP; Table S3: TV, CBV and ADC values before and during immunotherapy, in MGMT hyper- or un-methylated tumors.
