# Supplementary material for: Advanced MRI Assessment during Dendritic Cell Immunotherapy Added to Standard Treatment against Glioblastoma
Source: J Clin Med. 2019 Nov 17;8(11):2007. doi: 10.3390/jcm8112007 (PMC6912338; doi:10.3390/jcm8112007)
Supplement: Supplementary file 1 [file jcm-08-02007-s001.pdf]

Table S1. Normalized CBV and ADC values before and during immunotherapy.

	High/LowNK	aMRI-T0	aMRI-T2	aMRI-T4	aMRI-T12
**$rCBV_{mean}$**	HighNK	3.53 (0.56–9.45)	2.42 (0.6–8.66)	3.54 (0.76–7.97)	2.25 (0.7–4.8)
	LowNK	3.83 (2.25–7.51)	3.46 (1.51–5.89)	3.73 (1.08–4.98)	-
**$rCBV_{max}$**	HighNK	5.29 (0.56–10.55)	3.29 (0.92–14.14)	3.79 (0.76–10,04)	2.6 (0.71–6.89)
	LowNK	5.25 (2.27–9,69)	3.91 (3.46–9.28)	4.85 (1.08–6.82)	-
**$rADC_{mean}$**	HighNK	1.24 (0.92–1.53) [a]	1.06 (0.76–1.51) [b]	1.08 (0.83–1.62) [c]	1.13 (0.94–1.6)
	LowNK	1.02 (0.81–2.6)	1.18 (0.85–1.95)	1.1 (0.84–1.26)	1.02 (0.81–2.6)
**$rADC_{min}$**	HighNK	1.15 (0.82–1.47) [d]	1.01 (0.76–1.51) [e]	0.97 (0.67–1.34) [f]	1.02 (0.85–1.32)
	LowNK	0.97 (0.69–1.61)	1 (0.82–1.47)	1.02 (0.85–1.32)	

Note: a-b, p = 0.02; a-c, p = 0.023; d-e, p = 0.003; d-f, p = 0.005.

Table S2. CBV and ADC values with range observed two months before, during and two months after PsP.

	**MRI_{prePsP}**	**MRI_{PsP}**	**$MRI_{postPsP}$**	**$\Delta(MRI_{PsP}-MRI_{prePsP})$**	**$\Delta(MRI_{postPsP}-MRI_{PsP})$**
**$rCBV_{mean}$**	4.38 (0.99–8,66)	3.5 (0.86–6.59)	3.7 (2.15–4.61)	−1.08 (-4.75–2.59)	0.23 (−1.39–3.75)
**$rCBV_{max}$**	5.6 (1.85–14.14)	3.91 (0.75–10.04)	4.31 (2.97–7.63)	−2.64 (-5.35–3.68)	0.4 (−1.91–6.88)
**$rADC_{mean}$**	1.15 (1.01–1.43)	1.12 (0.97–1.62)	1.12 (0.97–2.11)	−0.05 (−0.3–0.22)	−0.07 (−0.25–1.14)
**$rADC_{min}$**	1.1 (0.81–1.43)	1.01 (0.82–1.34)	1.1 (0.88 -1.01)	−0.09 (−0.27–0.2)	−0.01(−0.22–1.18)

Table S3. TV, CBV and ADC values before and during immunotherapy, in MGMT hyper- or un-methylated tumors.

	MGMT status	aMRI-T0	aMRI-T2	aMRI-T4	aMRI-T12
Median TV (cm^3)	MGMT Hypermeth.	3.2 (0–11.1)	2.48 (0–38.34)	1.8 (0–7.5) [e]	3 (0–8.3)
	No MGMT Hypermeth. (15pts.)	5.2 (0.3–25.82)	6.92 (0.9–27)	5.85 (0.2–20.72) [f]	1.8 (0–7.51)
$rCBV_{mean}$	MGMT Hypermeth.	4.1 (0.56–7.51)	2.21 (0.6–4.21) [a]	1.93 (0.85–3.58)	2.25 (0.7–2.64)
	No MGMT Hypermeth. (15pts.)	3.69 (1.26–9.45)	3.46 (1.51–8.66) [b]	3.76 (0.76–7.97)	1.93 (0.85–3.58)
$rCBV_{max}$	MGMT Hypermeth.	1.49 (1.1–2.1)	2.63 (0.92–14.14) [c]	2.39 (0.94–5.53)	2.78 (0.71–2.85)
	No MGMT Hypermeth. (15pts.)	5.25 (1.94–10.55)	3.9 (1.81–14.14) [d]	4.86 (0.74–10.04)	2.39 (0.94–5.53)
$rADC_{mean}$	MGMT Hypermeth.	1.13 (1.02–1.47)	1.11(0.76–1.34)	1.17 (0.96–1.46)	1.08 (0.94–1.6)
	No MGMT Hypermeth. (15pts.)	1.15 (0.81–2.6)	1.12 (0.83–1.95)	1.04 (0.83–1.62)	1.17 (0.46–1.48)
$rADC_{min}$	MGMT Hypermeth.	1.08 (0.84–2.75)	1.01 (0.67–1.25	0.96 (0.83–1.31)	0.96 (0.85–1.31)
	No MGMT Hypermeth. (15pts.)	1.13 (0.69–1.61)	1.01 (0.81–1.47)	1 (0.67–1.34)	0.96 (0.83–1.31)
$ADC_{skewness}$	MGMT Hypermeth.	1.76 (0.99–3.15)	1.85 (0.84–2.75)	1.71 (0.71–2.35)	2.42(1.07–4.47)
	No MGMT Hypermeth. (15pts.)	1.68 (0.85–2.44)	1.69 (0.93–2.37)	1.69 (1.18–2.21)	1.49 (1.1–2.1)
ADC_{mode} (10^{-3} mm^2/s)	MGMT Hypermeth.	0.9 (0.3–2)	1 (0.3–1.3)	0.7 (0.2–1.3)	1.1 (0.1–2)
	No MGMT Hypermeth. (15pts.)	1.0 (0.4–1.9)	1.2 (0.6–1.8)	1.0 (0.2–1.7)	1.7 (1.1–2.1)
ADC_{mean} (10^{-3} mm^2/s)	MGMT Hypermeth.	1.4 (1–1.7)	1.4 (1.3–1.7)	1.3 (0.9–1.7)	1.2 (0.9–1.3)
	No MGMT Hypermeth. (15pts.)	1.3 (1.1–1.7)	1.4 (1.3–1.7)	1.5 (1–1.8)	1.5 (1.2–1.9)

Note: a-b, p = 0.035; c-d, p = 0.035; e-f, p = 0.046.

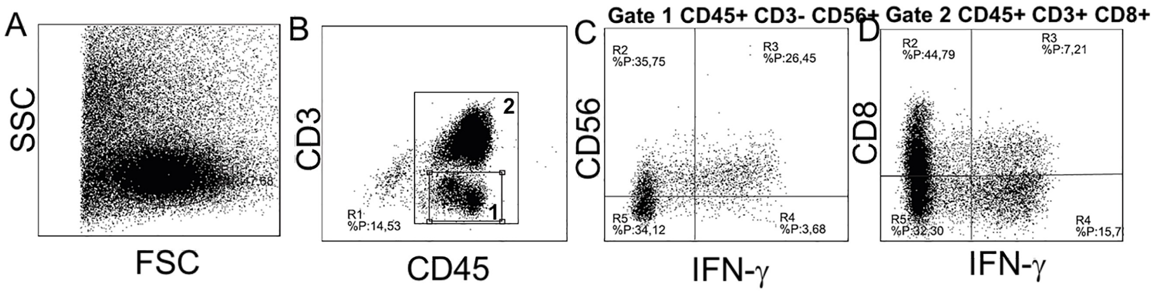

Figure S1. Flow cytometry assessment of active NK cells and CD8+ T cells; Representative dot plots show: (**A**) peripheral blood lymphocytes (PBLs) identified using Forward versus Side Scatter (FSC and SSC), based on size and granularity (complexity); (**B**) the gating strategy used to identify (1) CD45+ CD3- and (2) CD3+ CD45+ subset used to detect (**C**) CD56+ NK cells (Gate 1) and (**D**) CD8+ T cells (Gate 2) intracellularly stained with anti-IFN-g antibody.

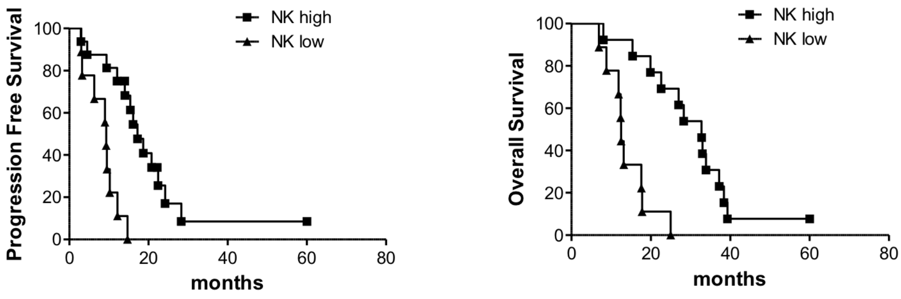

Figure S2. Meier curves display a significant correlation between high NK V/B ratio and prolonged survival; *** means p<0.001.
